# Zinc binding regulates amyloid-like aggregation of GAPR-1

**DOI:** 10.1042/BSR20182345

**Published:** 2019-02-12

**Authors:** Jie Sheng, Nick K. Olrichs, Willie J. Geerts, Xueyi Li, Ashfaq Ur Rehman, Barend M. Gadella, Dora V. Kaloyanova, J. Bernd Helms

**Affiliations:** 1 Department of Biochemistry and Cell Biology, Faculty of Veterinary Medicine, Utrecht University, Utrecht, the Netherlands; 2 Biomolecular Imaging, Bijvoet Center, Utrecht University, Utrecht, the Netherlands; 3 School of Pharmacy, Shanghai Jiao Tong University, Shanghai, China; 4 Department of Neurology, Massachusetts General Hospital and Harvard Medical School, Charlestown, Boston, MA, U.S.A.; 5 Department of Bioinformatics and Biostatistics, Shanghai Jiao Tong University, Shanghai, China

**Keywords:** Amyloid, CRISP, CAP superfamily, GAPR-1, Heparin, Zinc

## Abstract

Members of the CAP superfamily (Cysteine-rich secretory proteins, Antigen 5, and Pathogenesis-related 1 proteins) are characterized by the presence of a CAP domain that is defined by four sequence motifs and a highly conserved tertiary structure. A common structure–function relationship for this domain is hitherto unknown. A characteristic of several CAP proteins is their formation of amyloid-like structures in the presence of lipids. Here we investigate the structural modulation of Golgi-Associated plant Pathogenesis Related protein 1 (GAPR-1) by known interactors of the CAP domain, preceding amyloid-like aggregation. Using isothermal titration calorimetry (ITC), we demonstrate that GAPR-1 binds zinc ions. Zn^2+^ binding causes a slight but significant conformational change as revealed by CD, tryptophan fluorescence, and trypsin digestion. The Zn^2+^-induced conformational change was required for the formation of GAPR-1 oligomers and amyloid-like assemblies in the presence of heparin, as shown by ThT fluorescence and TEM. Molecular dynamics simulations show binding of Zn^2+^ to His^54^ and His^103^. Mutation of these two highly conserved residues resulted in strongly diminished amyloid-like aggregation. Finally, we show that proteins from the cysteine-rich secretory protein (CRISP) subfamily are also able to form ThT-positive structures *in vitro* in a heparin- and Zn^2+^-dependent manner, suggesting that oligomerization regulated by metal ions could be a common structural property of the CAP domain.

## Introduction

Golgi-Associated plant Pathogenesis Related protein 1 (GAPR-1), also known as GLIPR-2, is a mammalian protein that mainly localizes to lipid-enriched microdomains at the cytosolic leaflet of Golgi membranes [1]. GAPR-1 acts as a negative regulator of autophagy via the interaction with the essential autophagy effector Beclin 1. GAPR-1 retains Beclin 1 at the Golgi apparatus, thereby interfering with autophagy initiation [2]. GAPR-1 was also found to regulate type I interferon signaling activation in response to Toll-like receptor 4 [3].

GAPR-1 has a high affinity for membranes containing negatively charged lipids. We previously showed that membrane-bound GAPR-1 has a tendency to form homodimers, both on liposomes and on Golgi membranes [4,5]. Moreover, prolonged incubation with negatively charged liposomes resulted in the formation of amyloid-like fibrils [6]. GAPR-1 has been associated with amyloid-related diseases. GAPR-1 was found to be enriched in sites of induced neurodegeneration in rat hippocampus as well as in the insoluble proteome of multiple sclerosis (MS) patients [7,8] and was proposed to regulate the development of diabetic neuropathy [9]. Whether these pathologies are related to the amyloidogenic properties of GAPR-1, however, remains to be investigated.

The structural determinants for the amyloidogenic propensity of GAPR-1 are still unknown. Natively folded GAPR-1 binds the amyloid oligomer-specific antibody A11 and its presence was shown to inhibit amyloid β (Aβ) aggregation into amyloid fibrils, indicating an intrinsic amyloid-related structure [6]. In this respect, it is interesting to note that the crystal structure of GAPR-1 displays a dimeric arrangement with a near continuous β-sheet that extends beyond the monomeric subunits [5]. This suggests that subtle structural changes might be sufficient to induce amyloid formation. Indeed, upon membrane binding, oligomerization and fibrillation of GAPR-1 are rapidly initiated and enhanced by several factors including cholesterol [6].

GAPR-1 belongs to the CAP (Cysteine-rich secretory proteins, Antigen 5, and Pathogenesis-related 1) superfamily of proteins, members of which are found in a widespread variety of biological species with a remarkable diversity in functions [10]. Characteristic for CAP superfamily members is the presence of a CAP domain (or Sperm Coating Protein (SCP) domain) with four signature sequence motifs and a highly conserved tertiary structure with a unique α-β-α sandwich fold. Family members have an extension or an additional domain predominantly at the C-terminus of the CAP domain.

The role of the CAP domain in relation to the high diversity of functions of CAP proteins is still poorly understood. General functions that have been proposed include: (i) protease activity involving highly conserved catalytic amino acids at the dimer interface [5], (ii) lipid binding to a central cavity [11,12], and (iii) structural domain, regulating protein oligomerization [6,13]. Each of these functions requires structural flexibility of the CAP domain but so far, this has not been described. Here we focussed on the structural consequences of known interactors with the CAP domain. For several CAP proteins, it has been shown that metal binding is critical for specific functions [14–17] and that two highly conserved histidines of the CAP tetrad co-ordinate Zn^2+^ or other divalent metal ions. GAPR-1 consists almost exclusively of a CAP domain and the GAPR-1 subfamily was recently described to be the earliest evolutionary ancestor within the CAP superfamily [18]. GAPR-1 is therefore a suitable model protein to study the CAP domain structure–function relationship. In the present study, we show that GAPR-1 specifically binds zinc ions and that the formation of GAPR-1 oligomers and amyloid-like structures in the presence of heparin is regulated by zinc binding. We also provide evidence that metal-regulated oligomerization could be a common property of the CAP domain within the superfamily.

## Materials and methods

### Reagents

Heparin was purchased from Santa Cruz Biotechnology (Heidelberg, Germany); mouse CRISP2 (cysteine-rich secretory protein) (UniProtKD ID: P16563), human CRISP3 (UniProtKD ID: P54108), and mouse CRISP4 (UniProtKD ID: Q9D259) from R&D Systems (Minneapolis, U.S.A.); trypsin from Thermo Fisher Scientific (Eindhoven, the Netherlands); Thioflavin T (ThT), ZnCl_2_, ZnSO_4_, and EDTA from Sigma–Aldrich (St. Louis, U.S.A.).

### Plasmids

pQE60-GAPR-1 WT plasmid was described before [5,19]. pQE60-GAPR-1 H54A and pQE60-GAPR-1 H103A mutants were generated by site-directed mutagenesis using the following mutagenic primers. Altered sequences are shown in bold in Table 1. Mutations in GAPR-1 were verified by DNA sequencing (Baseclear, Leiden, the Netherlands).

**Table 1:** Primers used for site-directed mutagenesis

	Forward	Reverse
H54A	5′-gaggatcctcaag**gcc**agcccggagtcc-3′	5′-ggactccgggct**ggc**cttgaggatcctc-3′
H103A	5-cggggactgga**gcc**ttcacgccatg-3′	5′-catggccgtcaa**ggc**tccagtccccg-3′

### Protein expression and purification

Both wild-type (WT) and mutant GAPR-1 (UniProtKD ID: Q9H4G4) expression and purification have been described before [5,19]. Briefly, the protein was overexpressed in *Escherichia coli* XL-1 Blue host cells and induced by 1 mM IPTG overnight at 18°C. After cell pelleting and homogenization with a high pressure homogenizer (Avestin, Mannheim, Germany), soluble proteins were collected by two-step centrifugation for 30 min at 14000×***g*** followed by 30 min at 100000×***g***. GAPR-1 was purified by cation exchange chromatography using SP Sepharose FF (GE Healthcare, U.S.A.) eluted with a linear gradient of 0–400 mM NaCl in 25 mM Tris, pH 8.0. The purity of the isolated proteins was confirmed by SDS/PAGE and Coomassie Blue staining.

### Isothermal titration calorimetry

Isothermal titration calorimetric (ITC) analysis was carried out with a low volume NanoITC (TA Instruments-Waters LLC, New Castle, DE). The 50-μl syringe contained 1.5 mM ZnSO_4_ prepared in buffer containing 25 mM Tris, 50 mM NaCl, pH 7.4 (NT-50), and the 200 μl cell contained 60 μM GAPR-1 in NT-50 buffer. All solutions were degassed under vacuum for 10 min before use. Titrations were incremental with 2 μl injections at 300-s intervals. Experiments were performed at 20°C. The instrumental default setting plots exothermic events in the upward direction. Data were analyzed using Nano Analyze software (TA Instruments-Waters LLC).

### CD

Measurements of 15 μM GAPR-1 in the presence of 0–100 μM Zn^2+^ in NT-50 buffer were performed on a JASCO J-810 spectropolarimeter (Jasco Co. Ltd., Tokyo, Japan), using a 1-mm path length quartz cuvette, 1 nm bandwidth, 0.2 nm resolution, 1 s response time, and a scan speed of 20 nm/min. Temperature was controlled with a Peltier device at 37°C. Measurements were repeated four times.

### Tryptophan fluorescence assay

Tryptophan fluorescence was measured on a CLARIOstar microplate reader (BMG Labtech, Germany) using a 280-nm excitation filter and the maximum emission was measured at 336 nm. Fluorescence intensities of GAPR-1 (30 μM) in the presence of increasing concentrations of Zn^2+^ (0–500 μM) were normalized to the initial fluorescence intensity of GAPR-1 in the absence of Zn^2+^.

### Trypsin digestion

A total of 30 μM GAPR-1 was incubated with increasing Zn^2+^ concentrations (0–500 μM) in a total volume of 20 μl NT-50 buffer at 37°C for 30 min, after which trypsin was added in a molar ratio of 1:50 (trypsin: GAPR-1) and incubated at 37°C for 30 min. The reaction was terminated by the addition of sample buffer. Protein samples were analyzed by SDS/PAGE and Western blot using five different peptide GAPR-1 antibodies as listed in Table 2.

**Table 2:** List of GAPR-1 antibodies used in Western blot analysis

	Peptide	Origin	Producer
Ab1 (N-term.)	^3^KSASKQFHNE^12^	Mouse mAb	Abmart (Shanghai, China)
Ab2	^40^QQYSEALAST^49^	Mouse mAb	Abmart (Shanghai, China)
Ab3	^53^KHSPESSRGQ^62^	Mouse mAb	Abmart (Shanghai, China)
Ab4	^81^YNFQQPGFTS^90^	Mouse mAb	Abmart (Shanghai, China)
Ab5 (C-term.)	^143^GFFEENVLPPKK^154^	Rabbit pAb	Described before [1]

### ThT fluorescence assay

Kinetics of zinc-induced GAPR-1 amyloid-like aggregation in presence of heparin was monitored by ThT fluorescence. Reaction mixtures contained 15 μM GAPR-1, 37.5 μM heparin, and 50 μM ThT with or without 100 μM Zn^2+^ in NT-50 buffer and were incubated in sealed 96-well plates (Flat Clear Bottom Black Polystyrene, Corning, U.S.A.) at 37°C for 24 h. Fluorescence was measured in a CLARIOstar microplate reader (BMG Labtech, Germany). ThT fluorescence emission was recorded at 488 nm after excitation at 449 nm with agitation before every measurement.

### Western blot analysis of GAPR-1 oligomerization

The formation of higher order structures by GAPR-1 was analyzed by Western blot. GAPR-1 was incubated with 37.5 µM heparin in the presence and absence of 100 µM Zn^2+^ in NT-50 buffer at 37°C. Aliquots were taken after 0, 2, 7, and 20 h, respectively, mixed with non-reducing Laemmli sample buffer, boiled for 5 min, and separated by SDS/PAGE. Proteins were transferred to 0.45 µm Nitrocellulose membranes (Amersham Protran GE Healthcare) by Western blotting at 90 V for 1 h and visualized using rabbit polyclonal anti-GAPR-1 antibody (see Table 2) and HRP-labeled goat anti-rabbit secondary antibodies with ECL detection.

### TEM

A total of 15 μM GAPR-1 was incubated with 37.5 μM heparin in the presence or absence of 100 μM Zn^2+^ at 37°C for 6 h, 18 h, and 3 days. Ten microliters of each prepared sample was placed on a 100-mesh glow discharged gold grid (Quantifoil Micro Tools GmbH, Jena, Germany). After 30 s, excess fluid was removed, and the grid was washed twice with ddH_2_O for 30 s each time. Afterward, the grid was negatively stained with 2% uranyl acetate in ddH_2_O twice for 15 and 30 s, respectively. After the excess fluid was removed from the grid, it was dried on air. Samples were viewed in Tecnai 20 LaB6 transmission electron microscope (FEI, Eindhoven, the Netherlands) at 200 kV. Images were recorded using a 4K square pixelEagle CCD camera (FEI, Eindhoven, the Netherlands).

### Prediction of the Zn^2+^-binding sites

Prediction studies were performed in two ways: (i) a protein–protein interaction network was built to predict the homologous structure for GAPR-1 via the online database STRING v10.5 (http://string.embl.de/). Three clusters of homologous proteins were retrieved, with or without crystallographic structures deposited in the Protein Data Bank (PDB) database (www.rcsb.org). In order to find the Zn^2+^ binding site, a total of three protein crystals were extracted from the clusters, one from each cluster. Protein superimposition was done via PyMol v1.7 (www.pymol.org), which led to PDB code: 3qr1 crystallized with Zn^2+^; (ii) the Zn^2+^ binding site was determined using the online ZincBinder program (http://proteininformatics.org/mkumar/znbinder/index.html) [20]. The threshold was set to default in support vector machine (SVM), which classifies the zinc and non-zinc binding sites in protein sequences. ZincBinder accuracy of results commonly measured by the quantity of True Positives (TP), True Negatives (TN), False Positives (FP), and False Negatives (FN). In the prediction system, the total prediction accuracy, Matthew’s correlation co-efficient (MCC), sensitivity and specificity were calculated by following equations:


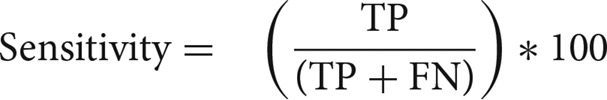



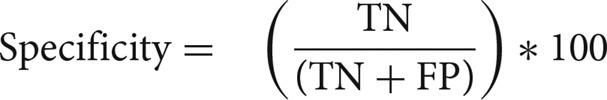



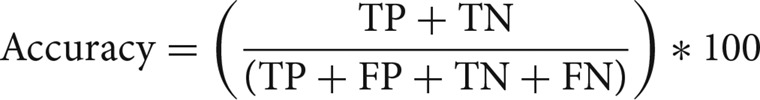



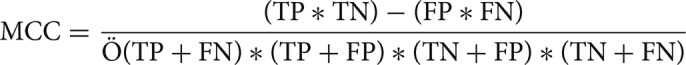


TP and TN are correctly predicted zinc metal binding sites and non-zinc metal binding sites, respectively. FP and FN are wrongly predicted zinc metal binding sites and non-zinc metal binding sites, respectively. The sites predicted by the ZincBinder program were cross-validated with the K_DEEP_ program for physiochemical properties, i.e. dissociation constant, the binding energy of Zn^2+^, and the binding efficiency in the protein.

## Results

### Zn^2+^ binding properties of GAPR-1

**Figure 1:**
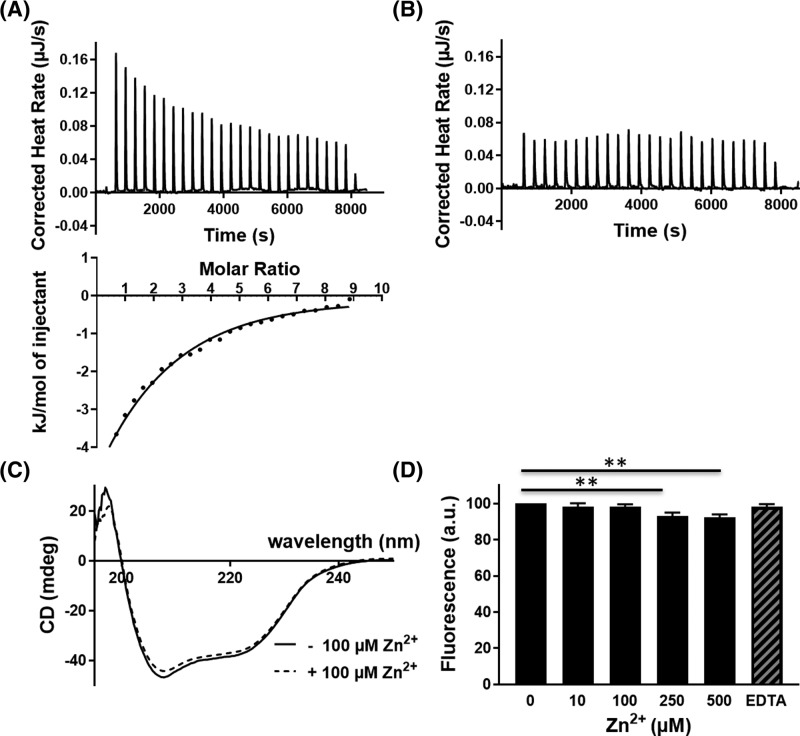
Zn^2+^ binding to GAPR-1 (**A**) ITC thermogram of Zn^2+^ titration into 60 μM GAPR-1. The upper panel represents the corrected heat rate. The lower panel shows the released heat compared with molar ratio of Zn^2+^ to GAPR-1. (**B**) ITC thermogram of Ca^2+^ titration into GAPR-1. (**C**) Far-UV CD spectra of 15 μM GAPR-1, recorded in the absence (solid line) and presence (dashed line) of 100 μM Zn^2+^. (**D**) Intrinsic tryptophan fluorescence of GAPR-1 in the absence or presence of Zn^2+^ (0–500 μM, as indicated). EDTA (1 mM) was subsequently added to the sample containing 500 μM Zn^2+^. Fluorescence intensities were normalized to the initial fluorescence intensity of GAPR-1 in the absence of Zn^2+^. The results represent the means (± S.D.) of three independent experiments. Statistical significance was determined by Student’s *t*-test; **P < 0.01.

Zinc ions have been shown to bind the CAP domain and therefore we investigated zinc binding to GAPR-1 using ITC. To this end, Zn^2+^ was repeatedly titrated into a GAPR-1 solution and the released heat was monitored by ITC (Figure 1A). A representative titration curve shows the repetitive release of energy, confirming the binding of Zn^2+^ to GAPR-1. Binding was specific for Zn^2+^ as energy release was not observed in the absence of metal ions (data not shown) nor in the presence of other metal ions, such as Ca^2+^ (Figure 1B). The thermodynamic parameters of GAPR-1–Zn^2+^ interaction were determined from the binding isotherm. The apparent dissociation constant is in the micromolar range (*K*_d_ = 76 ± 12 µM) with *n*=1.6 ± 0.3 (Figure 1A, bottom panel).

Next, we examined the effect of zinc binding on possible structural rearrangement(s) of GAPR-1 by CD spectroscopy. In the absence of zinc ions, a typical far-UV CD spectrum of GAPR-1 was observed with two minima at 207 and 222  nm, consistent with the predominant presence of α-helical elements (Figure 1C). When 15 μM GAPR-1 was incubated with up to 100 μM Zn^2+^ at 37°C, no significant changes in the far-UV CD spectrum were observed, indicating that zinc binding does not cause any major changes in GAPR-1 secondary structural elements. To confirm this finding, we also monitored the intrinsic tryptophan fluorescence of GAPR-1 upon the addition of zinc. Intrinsic tryptophan fluorescence has been used extensively to monitor small conformational changes within a protein [21]. GAPR-1 contains three tryptophan residues and recombinant GAPR-1 in solution emits light at 336 nm upon exposure to UV light. No shift in tryptophan emission maximum was observed upon addition of 100 μM Zn^2+^ (not shown). Increasing the ZnCl_2_ concentration did, however, cause a slight reduction in the emission intensity (Figure 1D). The changes were reversed upon addition of excess EDTA (Figure 1D), confirming that these effects were zinc-induced and reversible. In line with previous structural studies on other metal-bound CAP superfamily proteins, the moderate quenching of GAPR-1 intrinsic fluorescence combined with the CD data suggest no major structural rearrangements upon zinc binding.

### Zn^2+^ binding enhances susceptibility of GAPR-1 to trypsin digestion

**Figure 2:**
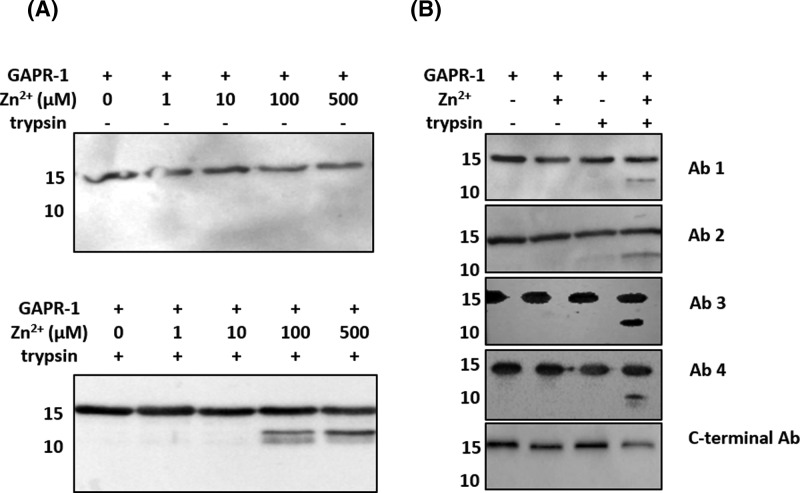
Zn^2+^ binding induces a conformational change in GAPR-1 (**A**) Western blot analysis of limited trypsin digestion of GAPR-1 using N-terminal GAPR-1 antibody. GAPR-1 (30 μM) was incubated in the presence of Zn^2+^ (0–500 μM) at 37°C for 30 min, followed by incubation without (upper panel) or with (lower panel) trypsin in the molar ratio of 1:50 (trypsin: GAPR-1) for 30 min at 37°C. (**B**) Western blot analysis of limited trypsin digestion of GAPR-1 incubated with 100 μM Zn^2+^ using five different GAPR-1 antibodies (see Table 2, ‘Materials and methods’ section).

To further examine the effect of zinc binding on the GAPR-1 structure, we used limited trypsin proteolysis in combination with Western blot analysis. GAPR-1 was incubated with trypsin for 30 min at 37°C in the presence or absence of zinc. Despite the presence of many lysines and arginines, GAPR-1 in its apo-form is completely resistant to digestion under the conditions employed (Figure 2A). However, trypsin treatment after titration of GAPR-1 with Zn^2+^ resulted in the appearance of a digestion product of ∼12 kDa (Figure 2A). This suggests that a trypsin cleavage site buried within the protein structure becomes exposed upon zinc binding. To obtain information on the position of the cleavage site, different GAPR-1 antibodies were used (see ‘Materials and methods’ section, Table 2). All of the tested antibodies except the C-terminal antibody (Ab5) recognized the 12-kDa band (Figure 2B), suggesting that the most probable site of cleavage is in a cluster of three lysines (K110, 113, and 114). Inspection of the GAPR-1 crystal structure (PDB: 1SMB) revealed that these three lysine residues are located in a loop preceding the C-terminal β-hairpin (Supplementary Figure S1).

### Zn^2+^ mediates amyloid-like aggregation of GAPR-1 in the presence of heparin

**Figure 3:**
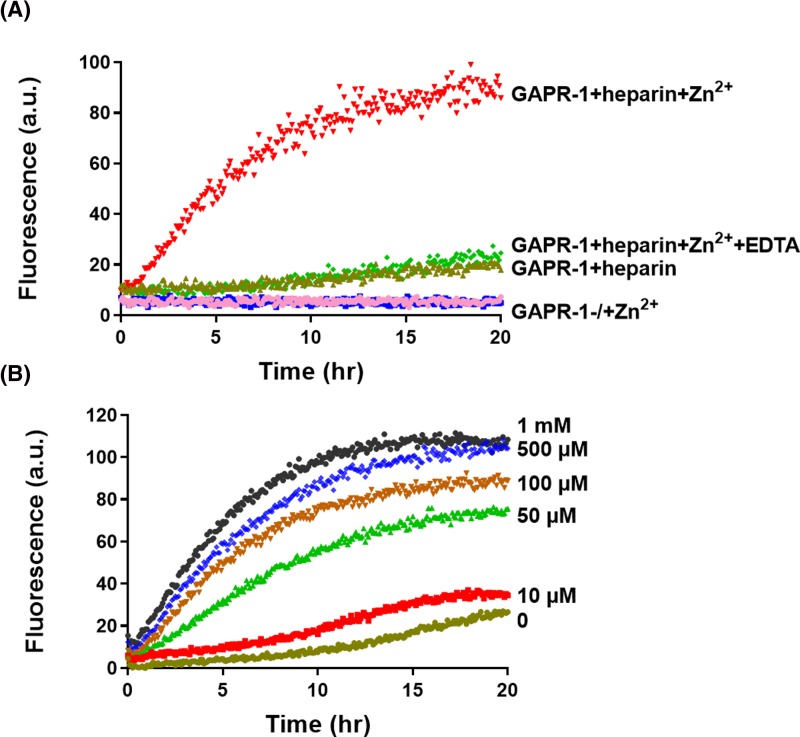
Zn^2+^ induces heparin-mediated GAPR-1 amyloid-like aggregation (**A**) Kinetics of ThT fluorescence enhancement of 15 μM GAPR-1 incubated with 37.5 μM heparin and 100 μM Zn^2+^. GAPR-1 incubated in the absence of heparin and/or Zn^2+^ and in the presence of 1 mM EDTA are shown as controls. (**B**) ThT fluorescence enhancement of 15 μM GAPR-1 incubated with 37.5 μM heparin in the presence of increasing concentrations of Zn^2+^ (0–1000 μM).

Given the subtle structural changes in the tertiary structure of GAPR-1 upon Zn^2+^ binding, we investigated whether Zn^2+^ binding affects the oligomerization/amyloid-like properties of GAPR-1. To this end, we used negatively charged polysaccharide heparin, which is commonly used to study aggregation induction and kinetics *in vitro* of amyloidogenic proteins [22,23]. Heparin serves as a platform to catalyze the seeding process as the first step in amyloid formation by increasing local protein concentrations. GAPR-1 was incubated with heparin in the presence or absence of zinc and ThT fluorescence was monitored over time (Figure 3A). When GAPR-1 was incubated both with heparin and with zinc ions, an immediate and rapid increase in ThT fluorescence intensity was observed. The fluorescence increase continued gradually over time reaching a plateau after approximately 12–15 h (Figure 3A). The immediate ThT increase indicates that oligomeric seeds are instantly formed upon binding of GAPR-1 to heparin in the presence of zinc ions. Furthermore, in the absence of Zn^2+^ the increase in ThT fluorescence after 20 h was negligible. In the absence of heparin or when Zn^2+^ was added in the presence of a molar excess of EDTA, ThT fluorescence did not increase significantly (Figure 3A). ThT fluorescence increased in a zinc concentration-dependent manner in the range of 0–1000 µM Zn^2+^ (Figure 3B). Taken together, these results indicate that zinc binding triggers amyloid-like aggregation of GAPR-1 in the presence of heparin.

**Figure 4:**
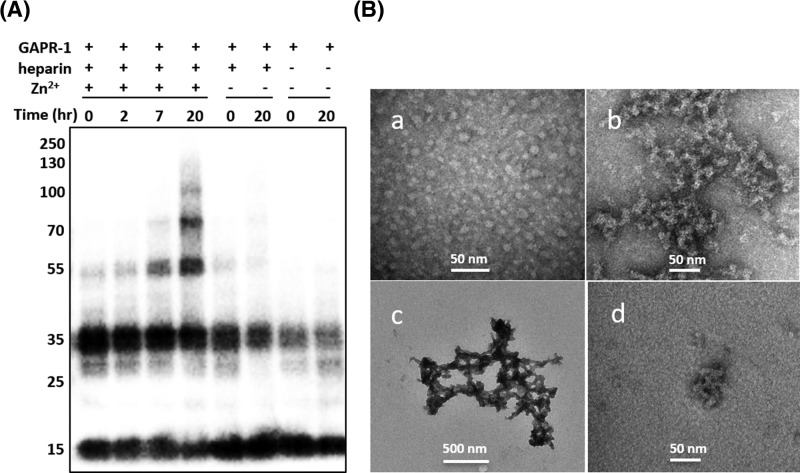
GAPR-1 forms oligomeric and amyloid-like structures in a zinc- and heparin-dependent manner (**A**) Western blot analysis of GAPR-1 (15 μM) after incubation in the absence or presence of 37.5 μM heparin and/or 100 μM Zn^2+^ at 37°C for 0, 2, 7, and 20 h, respectively. (**B**) Transmission electron micrographs of 15 μM GAPR-1 following incubation with 37.5 μM heparin and 100 μM Zn^2+^ at 37°C for 6 h (a), 18 h (b), and 3 days (**c**). GAPR-1 incubated with heparin in the absence of Zn^2+^ for 18 h (d). Scale bars represent 50 nm in panels (a,b,d), and 500 nm in panel (c).

The formation of higher order structures by GAPR-1 was confirmed by Western blot analysis. GAPR-1 was incubated at 37°C with heparin in the presence and absence of 100 µM Zn^2+^ and aliquots were taken after 0, 2, 7, and 20 h, respectively. Only in samples containing both heparin and Zn^2+^, an increasing amount of high molecular weight products became visible over time (Figure 4A). In the absence of heparin and/or zinc, only monomers and dimers were detected before and after 20 h incubation (Figure 4A).

GAPR-1 oligomeric/amyloid-like structures induced by Zn^2+^ and heparin were visualized by TEM. After 6 h of incubation, mainly small and spherical oligomers of approximately 5–10 nm in diameter were observed (Figure 4B(a)). Prolonged incubation of 18 or 72 h resulted in the formation of large amyloid-like aggregates of approximately 0.5 μm up to several μm (Figure 4B(b,c), respectively). GAPR-1 incubated with heparin in the absence of zinc showed small irregular clusters with diameters of approximately 50–100 nm (Figure 4B(d)). Aggregates were absent from TEM images of GAPR-1 and heparin controls, respectively (not shown).

### Putative metal ion binding site of GAPR-1

**Figure 5:**
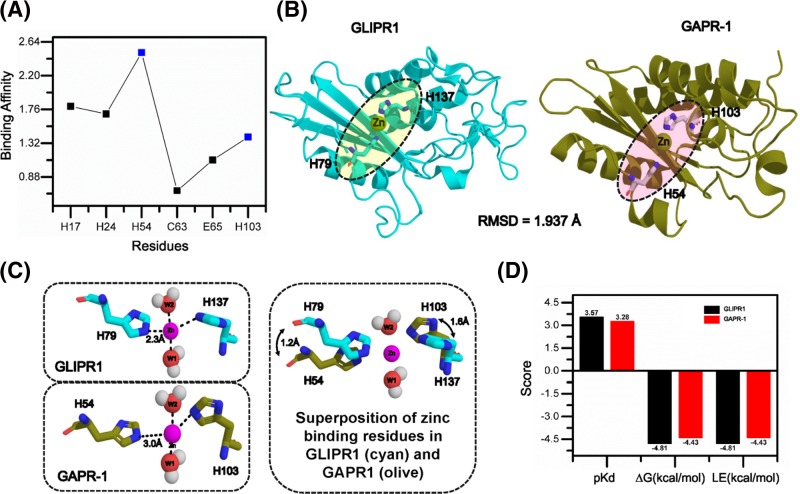
Co-ordination of zinc to GAPR-1 by His54 and His103 (**A**) Zn^2+^ binding site was predicted via ZincBinder. Zn^2+^ binding favors a tetrahedral geometry hierarchy and each residue in the potential binding site was analyzed for binding affinity. (**B**) Comparison of the 3D structure of GAPR-1 with the predicted Zn^2+^-binding site to the structure of Zn^2+^-bound GLIPR1. The RMSD between the crystals of Zn^2+^-bound GLIPR1 and GAPR-1 was less than 1.937 Å, indicative of a high similarity. (**C**) Comparison of the distance between the two histidine residues and Zn^2+^ in GLIPR1 and in GAPR-1. Left panel represents the tetrahedral geometry of Zn^2+^ binding in GLIPR1 and GAPR-1; right panel illustrates the superposition of the two Zn^2+^ binding residues in GLIPR1 and GAPR-1 and shows a rotation by 1.2 Å for His^54^ and 1.6 Å for His^103^ in GAPR-1 relative to His^79^ and His^137^ in GLIPR1, respectively. Each residue was labeled according to the crystallographic numbering. W represents a water molecule. (**D**) Physiochemical properties of zinc binding between GLIPR1 and GAPR-1 were compared. K_DEEP_ was utilized for analyzing the dissociation constant (pKd), the binding energy (ΔΔG), and the binding efficiency (LE). The corresponding score for GLIPR1 and GAPR-1 was plotted as a bar graph.

Zn^2+^ ions are co-ordinated by two highly conserved histidine residues in the CAP domain of snake venom CRISPs and GLIPR-1 [16,24,25]. Using bioinformatics tools calculating the minimal energy for metal ion binding, His^54^ and His^103^ were identified as the most likely amino acids involved in the co-ordination of Zn^2+^ at the GAPR-1 protein surface (Figure 5A). This identification is based on: (i) His^54^ and His^103^ were in close proximity, whereas all other predicted residues were distant from each other or buried (inaccessible for zinc binding) (His^17^ and His^24^ are indicated in Supplementary Figure S1); and (ii) His^54^ and His^103^ face each other in an opposite direction and possess enough binding affinity for Zn^2+^. The 3D structure of GAPR-1 docked with Zn^2+^ to the predicted site was compared with the crystal structure of Zn^2+^-bound GLIPR1 [25] (Figure 5B). The RMSD between the crystals of Zn^2+^-bound GLIPR1 and GAPR-1 was less than 1.937 Å, indicative of a high similarity. Zn^2+^ binding to GLIPR1 and GAPR-1 showed a tetrahedral geometry with two histidines and two water molecules (Figure 5C). The predicted Zn^2+^ binding residues in GAPR-1 (His^54^ and His^103^) were found to be in the same overall conformation as His^79^ and His137 in GLIPR1, except for a slight rotation of the corresponding histidine residues (Figure 5C, right panel). As a result, the distance between the two histidine residues in GLIPR1 (His^79^ and His^137^) and in GAPR-1 (His^54^ and His^103^) was 5.1 Å and 5.4 Å, respectively. The physiochemical properties, including dissociation constant (pKd), the binding energy (ΔG), and the binding efficiency (LE), was similar in zinc binding between GLIPR1 and GAPR-1 as shown in Figure 5D.

**Figure 6:**
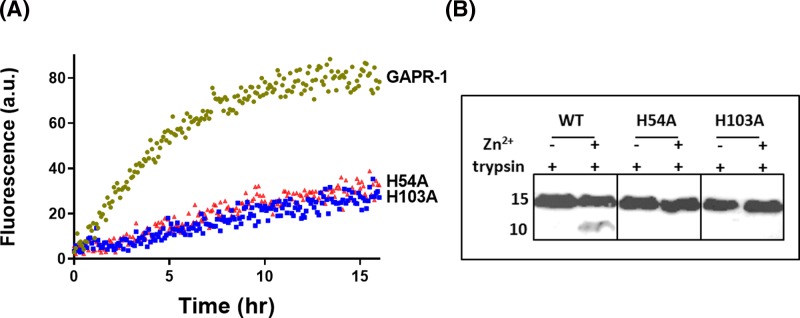
Mutation of the putative GAPR-1 metal-binding pocket affects amyloid formation (**A**) Kinetics of ThT fluorescence enhancement of 15 μM GAPR-1 WT, H54A or H103A, respectively, incubated with 5 μM heparin and 100 μM Zn^2+^ at 37°C. (**B**) Western blot analysis of limited trypsin digestion of GAPR-1 WT, H54A and H103A incubated with 100 μM Zn^2+^, using N-terminal GAPR-1 antibody.

To confirm the bioinformatics predictions on the involvement of H54A and H103A in Zn^2+^ binding and amyloid-like aggregation, we constructed two GAPR-1 point mutants in which either one of these conserved histidines was mutated to alanine (H54A and H103A). ThT fluorescence assay in the presence of heparin was performed to assess whether mutations in the putative metal binding pocket compromised zinc-induced amyloid-like aggregation. Figure 6A shows that for both H54A and H103A ThT fluorescence increase was severely diminished when incubated with heparin in the presence of Zn^2+^. Moreover, no trypsin cleavage was observed in the presence of Zn^2+^ for these mutants (Figure 6B). These results confirm the involvement of the two conserved histidines in zinc binding. At this stage, however, we cannot exclude the possibility that other residues are also involved in zinc binding.

**Figure 7:**
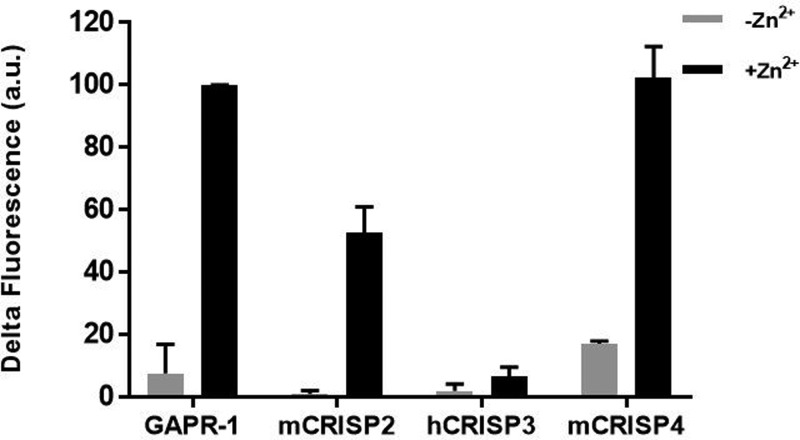
Zn^2+^ induces mouse CRISP2 and mouse CRISP4 amyloid formation A total of 2.5 μM GAPR-1, mouse CRISP2, human CRISP3 or mouse CRISP4 was incubated with 6.25 μM heparin in absence and presence of 100 μM Zn^2+^ at 37°C for 20 h. ThT fluorescence assay was performed and the ThT fluorescence was expressed as the difference between the end point and the starting point (Δ fluorescence). The results represent the means (± S.D.) of three independent experiments.

In sequence alignments with other family members, H54A and H103A of GAPR-1 align with two highly conserved histidines on either side of the groove of the CAP domain [10,26]. Given the highly conserved nature of the histidine binding site for Zn^2+^ binding, our findings suggest that zinc-dependent formation of amyloid-like structures may be a common property of other CAP proteins as well. We tested various proteins from the CRISP subfamily for amyloidogenic properties by monitoring ThT fluorescence. Recombinant mouse CRISP2, mouse CRISP4, and human CRISP3, respectively, were incubated with heparin in the presence or absence of Zn^2+^ and ThT fluorescence was monitored for 20 h. Figure 7 shows that for both mCRISP2 and mCRISP4, a Zn^2+^-dependent increase in ThT fluorescence occurred over time, whereas for hCRISP3 with or without Zn^2+^ no significant increase was observed.

## Discussion

Zinc binding was previously shown to be required for specific functions of CAP protein family members. For example, Natrin, a CAP protein present in snake venom, modulates inflammation through transcriptional regulation of vascular cell adhesion proteins in a Zn^2+^- and heparan sulphate-dependent manner [16]. Also, oligomeric complexes of Zn^2+^-bound CRISP1 are involved in association with sperm during epididymal maturation [15].

In the present study, we identify GAPR-1 as a zinc-binding protein and demonstrate the role of zinc in the regulation of the formation of amyloid-like structures by GAPR-1 in the presence of heparin. Zinc ions play a crucial and complex role in the modulation of amyloidogenic aggregation pathways of many proteins and peptides including τ, Aβ, islet amyloid polypeptide (IAPP), transthyretin (TTR), and superoxide dismutase 1 (SOD1) [27–30]. Zinc binding can cause conformational changes or stabilize certain molecular orientations which in turn can either trigger, alter, or inhibit the formation of cytotoxic amyloid structures [27,31–33]. In recent years also, zinc-dependent functional amyloids have been discovered [34,35]. Our data presented here provide some clues on the molecular mechanism of how zinc regulates the propensity of GAPR-1 to form amyloid-like assemblies in the presence of heparin. Zinc binding does not result in a major structural rearrangement as determined by CD spectroscopy and tryptophan fluorescence. In agreement with our observations, crystal structures of other CAP superfamily members in complex with Zn^2+^ also did not reveal major conformational changes within the CAP domain [16,24,25]. However, the increased susceptibility of GAPR-1 to trypsin digestion clearly demonstrates that zinc binding leads to a (slight) structural change that results in an exposure of a cleavage site in the C-terminal part of the protein. The trypsin cleavage site is not in direct proximity of the zinc binding site. Flexibility in the C-terminal part of the CAP domain is of particular interest as it contains the conserved CAP1 and CAP2 motifs, which were identified by amyloid prediction algorithms as amyloid-prone sequences [13]. This therefore suggests that the amyloid-related features within the GAPR-1 native structure can be tweaked by external factors such as zinc ions.

Heparin is a glycosaminoglycan (GAG), a family of long unbranched polysaccharides consisting of disaccharide repeating subunits with a large number of sulphate groups. Heparin and other GAGs are intimately involved in modulating amyloid formation of many proteins [36] including α-synuclein [37], τ [38], Aβ [39,40], and TTR [41,42]. GAPR-1 has a high isoelectric point of 9.4 which allows interaction with the negatively charged heparin *in vitro*. Heparin binding to other CAP proteins has also been observed [14,16]. Electron microscopy shows the formation of small protein clusters when GAPR-1 is incubated with heparin alone, indicating that heparin could serve as a platform for GAPR-1 aggregation. However, without Zn^2+^ binding this does not result in the formation of ThT-positive amyloid-like structures. These data suggest that the small conformational change is required but not sufficient for subsequent amyloid-like aggregation. Additional to the conformational change, a protein-concentrating step is required to initiate or catalyze amyloid formation. Previously, we showed that oligomerization and fibrillation of GAPR-1 occurs spontaneously upon interaction with negatively charged liposomes without the necessity of metal ions [4]. The initial binding step based on electrostatic interactions of the positively charged GAPR-1 with both the negatively charged liposomes and heparin are likely to be comparable and reflect a concentration step using two different platforms. We suggest that hydrophobic interactions with the membrane stabilize structural rearrangements in the CAP domain, affecting the structural stability in such a way that amyloid-prone regions become increasingly exposed over time. In the absence of membranes, other mechanisms such as Zn^2+^ binding can also induce structural rearrangements. Our studies identify a two-step mechanism in the amyloid-like aggregation of GAPR-1: (i) a concentration step. Different platforms (membranes, heparin) can serve this purpose and allow the formation of amyloid seeds; and (ii) a conformational change. Different molecules (lipids, Zn^2+^ ions) can serve this purpose and only a subtle conformational change is required. The exact molecular mechanism will be the subject of further studies.

There is growing evidence that di-/oligomerization of the CAP domain is important for the function of CAP superfamily members [5,15,43–45]. This suggests that the CAP domain could serve as a scaffold to localize and enhance the action of other domains in CAP proteins through oligomerization [13]. We show here that other CAP superfamily members with an additional C-terminal domain, i.e. mouse CRISP2 and CRISP4, also form ThT-positive structures in a zinc- and heparin-dependent manner. This strongly suggests that oligomerization and/or amyloid-like aggregation is a common property of the CAP domain. Although human CRISP3 did not show this characteristic in the presence of heparin and zinc, it does not rule out the possibility that other factors will trigger oligomerization of this particular protein. CAP family members are present in a wide variety of unique environments, each facing an entirely different spectrum of cell surface lipids, GAGs, or other potential activating agents. In addition, the extension of the CAP domain is different for every family member, allowing another level of regulation.

In conclusion, we propose that amyloid-like oligomerization is a common property of the CAP domain in CAP superfamily members and involved in regulating the diverse biological functions of the various family members.

## Supplementary Material

Supplementary Figure S1

## Abbreviations

Aβ: amyloid β
CAP: cysteine-rich secretory proteins, antigen 5, and pathogenesis-related 1
CD: circular dichroism
CRISP: cysteine-rich secretory protein
FN: false negative
FP: false positive
GAG: glycosaminoglycan
GAPR-1: Golgi-associated plant pathogenesis related protein 1
GLIPR1: glioma pathogenesis-related protein 1
ITC: isothermal titration calorimetry
PDB: Protein Data Bank
TEM: transmission electron microscopy
ThT: Thioflavin T
TN: true negative
TP: true positive
TTR: transthyretin
