# Supplementary figures and images for: Zinc binding regulates amyloid-like aggregation of GAPR-1
Source: Biosci Rep. 2019 Feb 12;39(2):BSR20182345. doi: 10.1042/BSR20182345 (PMC6900432; doi:10.1042/BSR20182345)

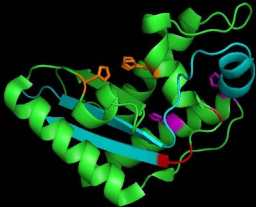

Supplement: Supplementary Figure S1 [file BSR-2018-2345_supp.pdf]
